# Characterization of Enterobacterales growing on selective CPE screening plates with a focus on non-carbapenemase-producing strains

**DOI:** 10.1128/spectrum.02079-24

**Published:** 2025-01-14

**Authors:** Reut Efrati Epchtien, Elizabeth Temkin, Mor N. Lurie-Weinberger, Ophir Kastel, Alona Keren-Paz, David Schwartz, Yehuda Carmeli

**Affiliations:** 1 National Institute for Antibiotic Resistance and Infection Control, Israel Ministry of Health, Tel Aviv, Israel; 2 Faculty of Medicine, Tel Aviv University 26745 , Tel Aviv-Yafo, Israel; JMI Laboratories, North Liberty, Iowa, USA

**Keywords:** Enterobacterales, screening, carbapenem resistance, beta-lactamase

## Abstract

Carbapenem-resistant Enterobacterales (CRE) are divided into two distinct groups: carbapenemase-producing (CPE) and non-carbapenemase-producing (non-CPE). The population of non-CPE growing on CPE selective plates during routine screening is usually not reported and is not well defined. This study aimed to characterize non-CPE isolates growing on those plates. Isolates were collected from two medical institutions in Israel between May and November 2022. Species identification and antibiotic susceptibility testing were performed using VITEK systems. Further analyses focused on *Klebsiella pneumoniae* and *Escherichia coli* which were the most common isolates. Meropenem MIC was determined by E-test. Fourier-transform infrared spectroscopy (FTIR) was used to analyze phenotypic similarity, and whole genome sequencing was conducted on a subset of non-CPE isolates. Of 260 isolates growing on selective CPE screening plates, 60.4% were non-CPE. The most common non-CP isolates were *K. pneumoniae* (65.6%) followed by *E. coli* (20.4%). Of the non-CP *K. pneumoniae* and *E. coli* isolates, 78.2% were susceptible to meropenem. All non-CP *K. pneumoniae* and *E. coli* isolates were multidrug-resistant (MDR) and belonged to multiple FTIR clusters. The 21 sequenced non-CPE isolates carried multiple mechanisms of resistance. Isolates had multiple beta-lactamases and all had penicillin-binding protein modifications and porin mutations; in meropenem-resistant *K. pneumoniae* isolates, both Ompk35 and Ompk36 were mutated. The majority of isolates growing on selective CPE screening plates are non-CPE but are MDR. Laboratory reporting of these MDR bacteria might be useful for guiding treatment and prophylaxis when indicated, as well as for infection control.

**IMPORTANCE:**

Selective screening plates for carbapenemase-producing Enterobacterales (CPE) are used to detect CPE carriers for infection control purposes. We characterized non-CPE isolates that grew on selective CPE screening plates, which are intended to filter them out. We found that 60% of isolates that grew on these plates were not CPE. They included both meropenem-susceptible and meropenem-resistant isolates and were multidrug-resistant with multiple resistance mechanisms. These test results, which are usually not reported by laboratories, may be clinically valuable.

## INTRODUCTION

Enterobacterales frequently cause infections acquired in hospitals as well as the community (1–3); infections that lead to an increase in morbidity and mortality (4, 5). Carbapenem-resistant Enterobacterales (CRE), including *Escherichia coli*, *Klebsiella pneumoniae*, and *Enterobacter* spp., are considered an “urgent threat” to public health by the US Centers for Disease Control and Prevention (5) and a “critical priority” for drug development by the World Health Organization (6).

CRE are divided, based on the mechanism of resistance, into two distinct groups: carbapenemase-producing (CPE) and non-CP Enterobacterales (non-CPE). CPE carry enzymes, such as *bla*_KPC_, *bla*_NDM_, *bla*_OXA-48-like_, and *bla*_VIM_, which hydrolyze carbapenems efficiently. These genes can transfer horizontally through mobile genetic elements such as plasmids or transposons (7). In contrast, non-CP CRE carry β-lactamases, such as AmpC or extended-spectrum β-lactamases (ESBL), which do not hydrolyze carbapenems efficiently (8). Nevertheless, hyperproduction of these enzymes, combined with reduced intracellular concentration of carbapenems (due to decreased outer membrane permeability mediated by porin loss or modification), leads to a carbapenem-resistant phenotype (9).

Screening to detect CPE carriers is an important measure to limit the spread of carbapenemases. Using selective screening agar plates with high sensitivity is desirable to enable the detection of CPE with low carbapenem MICs, such as Enterobacterales harboring OXA-48-like (10). The trade-off of high sensitivity is reduced specificity, leading to the growth of non-CPE (8).

The population of non-CPE growing on CPE selective plates is usually ignored during routine screening and is not well defined but may be of interest. In this study, we aimed to characterize non-CPE isolates growing on selective CPE screening plates.

We hypothesized that these non-CPE (i) are multidrug-resistant, (ii) represent heterogeneous species, clones, and resistance mechanisms, and (iii) include not only carbapenem-resistant but also carbapenem-susceptible strains (with elevated carbapenem MICs).

## MATERIALS AND METHODS

### Setting and sample

Isolates were collected from one acute care hospital and one post-acute care hospital in Israel between May and November 2022. All isolates were screening samples obtained by rectal swab. The sample consisted of 290 isolates from 239 unique patients: 187 non-CPE and 103 CPE.

### PCR technique

Isolates that grew on CHROMagar mSuperCARBA plates (HyLabs, Rehovot, Israel) underwent PCR for common carbapenemase genes (one multiplex for *bla*_KPC_ and *bla*_OXA-48-like_ and a second multiplex for *bla*_NDM_, *bla*_VIM_, and *bla*_IMI_). We used previously described primers for *bla*_VIM_ (11) and *bla*_IMI_ (12). We used newly designed primers for the other carbapenemase genes. For *bla_KPC_* forward/reverse: 5′-GACACACCCATCCGTTACG/GCATAGTCATTTGCCGTGC-3′, for *bla_OXA-48_* forward/reverse: 5′- CTGAACATAAATCACAGGGCGTAG/CGAGCCAGAAACTGTCTACATTGC-3′, and for *bla_NDM_* forward/reverse: 5′-CATTAGCCGCTGCATTGATGCT/TAGTGCTCAGTGTCGGCATCACC-3′. Amplification was carried out with the following thermal cycling conditions: 2 min at 95°C and 35 cycles of amplification consisting of 10 s at 95°C, 15 s at 60°C (for multiplex 1) or 62°C (for multiplex 2), and 7 s at 72°C.

### Classification of isolates as carbapenemase-producing or non-carbapenemase-producing

Following PCR, all isolates were tested for imipenem hydrolysis (β CARBA, Bio-Rad, Marnes-la-Coquette, France). Isolates that were positive by PCR and hydrolysis were classified as CPE. All others were defined as non-CPE. We confirmed that non-CPE were hydrolysis-negative using the modified carbapenem inactivation method (mCIM). Usually, hydrolysis-positive and PCR-negative strains are subjected to whole genome sequencing (WGS) to search for carbapenemases; there were no such strains in our sample.

### Identification and antibiotic susceptibility testing

Species identification for CPE was done by VITEK MS (bioMérieux, Marcy l’Etoile, France). Species identification and antibiotic susceptibility testing for non-CPE were done by VITEK 2 (bioMérieux). For *K. pneumoniae* and *E. coli*, meropenem MIC was determined by E-test (bioMérieux) using break points according to CLSI guidelines (13); each isolate was tested twice, and a third test was performed if the results were discordant. We defined multi-drug resistance (MDR) according to Magiorakos et al. (14).

### Fourier-transform infrared spectroscopy

To test our hypothesis that non-CPE represent heterogeneous clones, we analyzed the phenotypic similarity between non-CP *K. pneumoniae* and *E. coli* by Fourier-transform infrared spectroscopy (FTIR) using previously described methods (15). One isolate per patient was included. Isolates were grown at 35 ± 2°C on blood agar plates (HyLabs) for 24 h. Samples were prepared according to the IR Biotyper (Bruker, Leipzig, Germany) manufacturer’s instructions. We analyzed at least three replicates per sample using the Biotyper’s default settings. Spectra were analyzed by OPUS 7.5 software (Bruker), which generated a hierarchical cluster analysis and displayed it as a dendrogram, using the Pearson correlation coefficient option. We chose the cut-off values to define dendrogram clusters based on visual inspection and within the range recommended by the manufacturer.

### Whole genome sequencing

Based on the MIC and FTIR results, we chose 21 non-CPE isolates to undergo WGS: eight meropenem-resistant (mem-R) *K. pneumoniae* and four meropenem-susceptible (mem-S) *K. pneumoniae* that belonged to the same cluster as a mem-R isolate; and seven mem-R *E. coli* and two mem-S *E. coli* (one that belonged to the same cluster as a mem-R isolate and one with no resistant match). Samples were sequenced using Oxford Nanopore at SNPsaurus, Eugene, Oregon. ST type was determined using pubMLST (https://pubmlst.org). Antibiotic resistance genes (ARGs) were detected using ResFinder version 4.6.0 (https://cge.food.dtu.dk/services/ResFinder/).

### Protein analysis

Porin and penicillin-binding protein (PBP) encoding genes for non-CP *K. pneumoniae* and *E. coli* isolates were analyzed. Genes were detected by BLASTN and the six possible reading frames were manually assessed using Expasy for mutations causing protein truncation (https://www.expasy.org/).

### Statistical methods

We used an online calculator to perform a test of proportions comparing the proportions of CP and non-CP isolates that were mem-S (https://www.medcalc.org/calc/comparison_of_proportions.php).

## RESULTS

A total of 260 Enterobacterales growing on selective plates were collected for this study: 103 (39.6%) CPE and 157 (60.4%) non-CPE (Table 1). Among CPE, *E. coli* (55/103, 53.4%) and *K. pneumoniae* (18/103, 17.5%) were the most common isolates. The most common carbapenemase was OXA-48-like among *E. coli* and NDM among *K. pneumoniae*. Of the 157 non-CPE isolates, the vast majority were *K. pneumoniae* (103/157, 65.6%) followed by *E. coli* (32/157, 20.4%). For further analyses, we enriched the sample with 30 additional *E. coli* (for a total of 62 non-CP *E. coli*). All these 187 non-CPE isolates were MDR (Table S1).

**TABLE 1:** Distribution of species and carbapenemases among 260 Enterobacterales isolates

	Carbapenemase						
Species	Negative	Positive	KPC	NDM	OXA	VIM	>1
*Citrobacter braakii*	0	1	1				
*Citrobacter freundii*	1	3	2				1
*Citrobacter sedlakii*	1	0					
*Escherichia coli*	32	55	1	22	31		1
*Enterobacter aerogenes*	6	0					
*Enterobacter cloacae complex*	7	10		10			
*Enterobacter hormaechei*	0	8	1	5		2	
*Klebsiella aerogenes*	0	3	1		1		1
*Klebsiella oxytoca*	2	4	2	2			
*Klebsiella pneumoniae*	103	18	3	12	3		
*Lelliottia amnigena*	1	0					
*Morganella morganii*	0	1		1			
*Pantoea* spp.	1	0					
*Raoultella planticola*	1	0					
*Serratia fonticola*	1	0					
*Serratia marcescens*	1	0					
Total	157	103	11	52	35	2	3

MICs of 228 CP and non-CP *K. pneumoniae* and *E. coli* isolates are presented in Table 2 and Table S2. Among *E. coli*, 17/47 (36.2%) CP and 47/62 (75.7%) non-CP were mem-S (MIC <1, *P* < 0.001). Among *K. pneumoniae*, 1/16 (6.3%) CP and 82/103 (79.6%) non-CP were mem-S (*P* < 0.001). Half of the mem-S isolates (66/129, 51.2%) had elevated meropenem MICs within the susceptible range (>0.125).

**TABLE 2:** Carbapenem MIC distribution as determined by E-test of CP and non-CP *K. pneumoniae* and *E. coli* isolates^a^

	*K. pneumoniae*		*E. coli*	
Meropenem MIC (µg/mL)	Non-CP, *N* = 103 n (cumulative %)	CP, *N* = 16 n (cumulative %)	Non-CP, *N* = 62 n (cumulative %)	CP, *N* = 47 n (cumulative %)
0.032	3 (2.9)	0	2 (3.2)	0
0.064	3 (5.8)	0	8 (16.1)	0
0.125	36 (40.8)	0	11 (33.8)	3 (6.4)
0.25	19 (59.2)	0	15 (58)	5 (17)
0.5	13 (71.8)	0	9 (72.5)	5 (27.6)
1	8 (79.6)	1 (6.3)	2 (75.7)	4 (36.1)
2	6 (85.4)	0	4 (82.2)	5 (46.7)
4	6 (91.2)	0	4 (88.7)	5 (57.3)
8	3 (94.1)	2 (18.8)	4 (95.2)	3 (63.7)
12	1 (95.1)	0	0	0
16	3 (98)	0	1 (96.8)	2 (68)
≥32	1 (99)	13 (100)	0	15 (100)
Heteroresistance	1 (100)	0	2 (100)	0

^a^ MIC was unavailable for two CP *K*. *pneumoniae* and eight CP *E. coli*.

**Fig 1:**
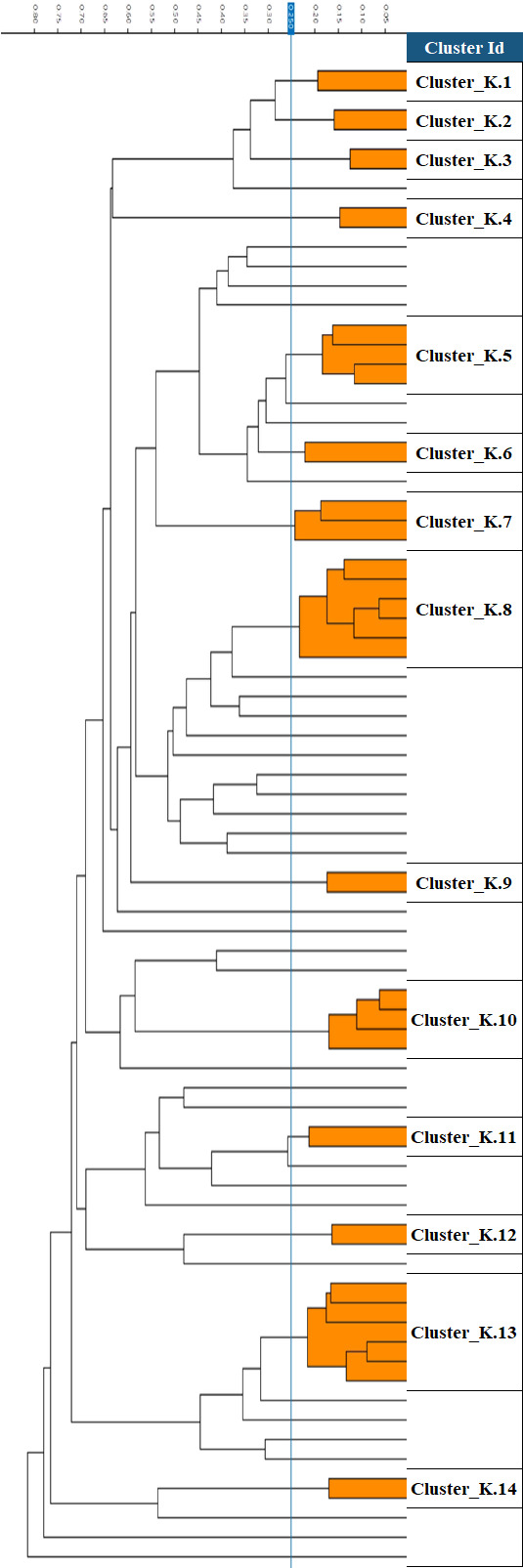
FTIR dendrogram of 77 non-carbapenemase-producing *K. pneumoniae*. Cut-off value of 0.25 is shown as a vertical blue line.

**Fig 2:**
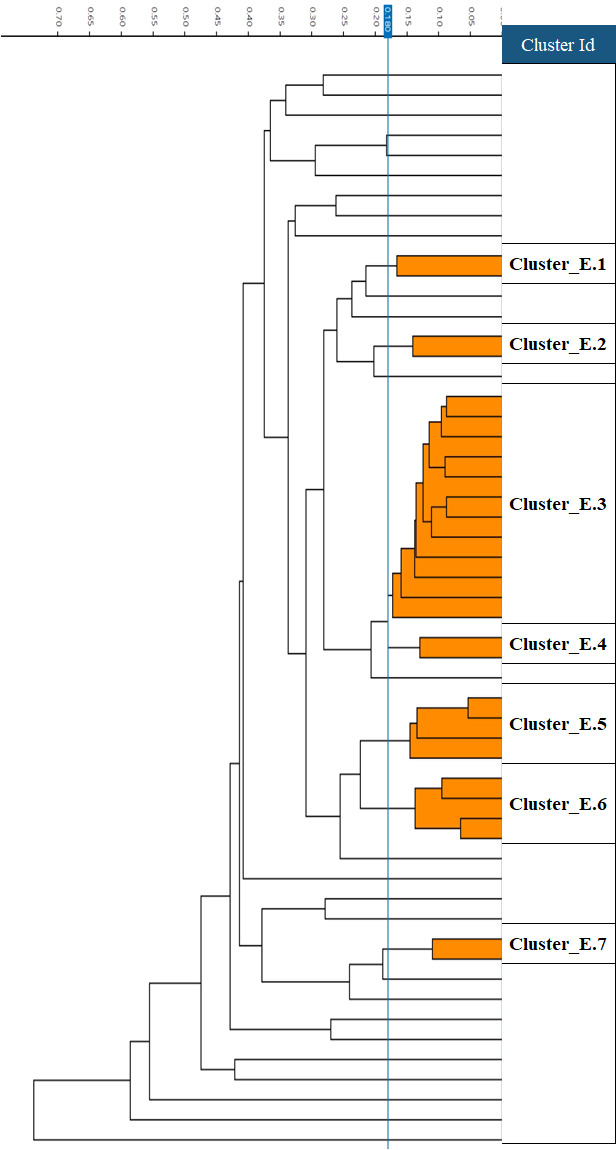
FTIR dendrogram of 54 non-carbapenemase-producing *E. coli*. Cut-off value of 0.18 is shown as a vertical blue line.

Next, we examined the phenotypic similarities of 77 non-CP *K. pneumoniae* and 54 non-CP *E. coli* isolates. (We excluded duplicate isolates from the same patient.) When analyzed by FTIR, the isolates of both species were mostly singletons or belonged to small clusters. Twelve (15.6%) of the 77 *K*. *pneumoniae* isolates formed two large clusters (>5 isolates), while the majority of the isolates belonged to 12 small clusters (2–5 isolates) and 37 singletons (Fig. 1). The 54 *E. coli* isolates formed one large cluster of 12 isolates (22.2%), six small clusters, and 26 singletons (Fig. 2). For *K. pneumoniae*, we observed four instances of mem-R and mem-S isolates clustering together, while for *E. coli*, only one such case was present (Table 3).

**TABLE 3:** FTIR clustering, MLST typing, porins, PBPs, and β-lactamases of sequenced non-carbapenemase-producing *K. pneumoniae* and *E. coli* isolates^*a*^

					MLST		Porins *K. pneumoniae*			Porins *E. coli*			PBPs			
Sample	Meropenem MIC	Bacteria	FTIR cluster	Total isolates in cluster (*N*)^*b*^	Achtman	Pasteur	Ompk35	Ompk36	Ompk37	OmpC	OmpF	LamB	PBP1	PBP2	PBP3	β-lactamases
6700988	8	*K. pneumoniae*	Cluster_K.1	2		2010	0	0	1				0	0	0	*bla*_CTX-M-15_, *bla*_OKP-A-9_
6700694	4	*K. pneumoniae*	Cluster_K.4	2		323	0	0	1				0	0	0	*bla*_CTX-M-15_, *bla*_SHV-99_, *bla*_TEM-206_
6700823	16	*K. pneumoniae*	Cluster_K.7	3		45	0	0	1				0	0	0	*bla*_SHV-1_, *bla*_TEM-1B_, *bla*_DHA-1_
6700745	0.125	*K. pneumoniae*	Cluster_K.7	3		45	0	1	1				0	0	0	*bla*_CTX-M-15_, *bla*_OXA-1_, *bla*_SHV_, *bla*_TEM-1B_
6700349	12	*K. pneumoniae*	Cluster_K.9	2		1774	0	0	1				0	0	0	*bla*_CTX-M-15_, *bla*_SHV-11_
6700362	0.125	*K. pneumoniae*	Cluster_K.9	2		1774	0	1	1				0	0	0	*bla*_CTX-M-15_, *bla*_SHV_
6700708	4	*K. pneumoniae*	Cluster_K.10	4		39	0	0	1				0	0	0	*bla*_CTX-M-15_, *bla*_SHV_, *bla*_TEM-1B_
6700958	0.25	*K. pneumoniae*	Cluster_K.10	4		39	0	1	1				0	0	0	*bla*_CTX-M-15_, *bla*_OXA-1_, *bla*_SHV_, *bla*_TEM-1B_
6700828	4	*K. pneumoniae*	Cluster_K.11	2		N/A^*c*^	0	0	1				0	0	0	*bla*_SHV-1_, *bla*_SHV-27_, *bla*_SHV-49_
6700792	0.25	*K. pneumoniae*	Cluster_K.11	2		15	0	1	1				0	0	0	*bla*_CTX-M-15_, *bla*_OXA-1_, *bla*_SHV_, *bla*_TEM-1B_
6700703–2	8	*K. pneumoniae*	Cluster_K.12	2		N/A	0	0	1				0	0	0	*bla*_CTX-M-15_, *bla*_OXA-1_, *bla*_SHV-72_, *bla*_TEM-1B_
6700899	16	*K. pneumoniae*	Singleton	0		N/A	0	0	0				0	0	0	*bla*_CTX-M-15_, *bla*_OXA-1_, *bla*_SHV-62_
6700890	8	*E. coli*	Cluster_E.1	2	405	N/A				0	0	0	0	0	0	*bla*_CTX-M-15_, *bla*_OXA-1_, *bla*_EC-8_
6701177	16	*E. coli*	Cluster_E.3	12	131	43				0	0	0	0	0	0	*bla*_CTX-M-15_, *bla*_OXA-1_
6700437	0.032	*E. coli*	Cluster_E.3	12	131	43				0	0	0	0	0	0	*bla*_CTX-M-15_, *bla*_OXA-1_
6701199	0.25	*E. coli*	Cluster_E.4	2	45	638				0	0	0	0	0	0	*bla*_EC-15_
6701402	4	*E. coli*	Cluster_E.5	4	131	N/A				0	0	0	0	0	0	*bla*_CTX-M-15_, *bla*_OXA-1_
6701403	4	*E. coli*	Cluster_E.5	4	N/A	43				0	0	0	0	0	0	*bla*_CTX-M-15_, *bla*_OXA-1_
6701347	8	*E. coli*	Cluster_E.6	4	131	43				0	0	0	0	0	0	*bla*_CTX-M-15_, *bla*_OXA-1_, *bla*_CMY-42_
6700970	8	*E. coli*	Cluster_E.6	4	5640	N/A				0	0	0	0	0	0	*bla*_CTX-M-15_, *bla*_OXA-1_
6701249	8	*E. coli*	Singleton	0	167	N/A				0	0	0	0	0	0	*bla*_CTX-M-15_, *bla*_OXA-1_, *bla*_EC-15_, *bla*_CMY-42_

^*a*^ Gray rectangles are pairs of meropenem-susceptible and meropenem-resistant isolates belonging to the same FTIR cluster and ST.

^*b*^ Includes isolates that were not sequenced: 0 = truncated and 1 = intact.

^*c*^ Indicates sequence type not available.

In order to correlate the FTIR results with MLST, examine the presence of ARGs, and examine differences in ARGs between mem-R and mem-S isolates, we selected 21 isolates for WGS analysis based on their meropenem susceptibility and FTIR clustering. For *K. pneumoniae,* MLST mostly correlated with the FTIR clustering, but in a few cases, FTIR results were more discriminative than MLST (Table 3). We sequenced seven mem-R isolates from seven different clusters (or singletons). Five belonged to different STs and two were unassigned. When we examined four mem-R and mem-S pairs belonging to the same cluster, in three of the pairs the mem-S and mem-R isolates belonged to the same ST. For *E. coli*, there was a poor correlation between clusters and STs. We sequenced seven mem-R isolates from five different clusters (or singletons). Three isolates from different clusters belonged to the same Achtman ST, and among 2 pairs of isolates from the same cluster, each member of the pair belonged to a different ST. There was one mem-R and mem-S pair belonging to the same cluster; both isolates belonged to the same ST.

**Fig 3:**
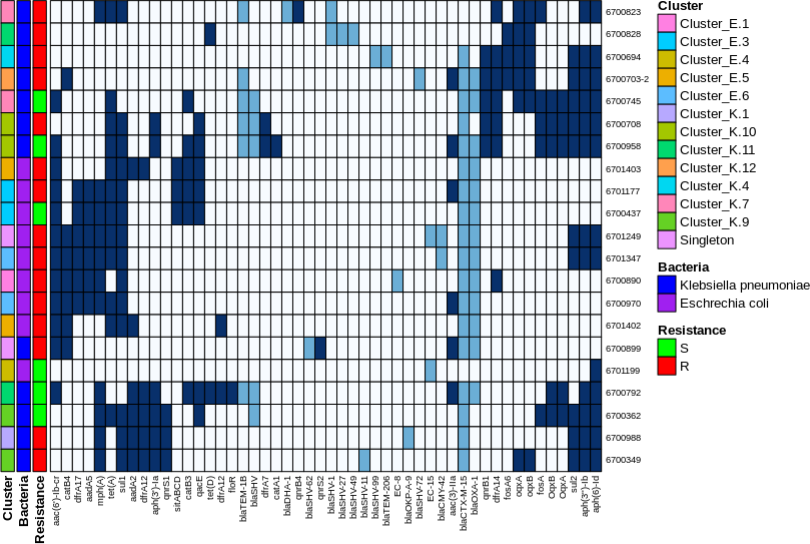
Heatmap depicting clustering of antibiotic resistance genes in non-carbapenemase-producing isolates. Blue squares indicate gene is present, light blue indicates β-lactamase gene is present, and white indicates gene is absent.

Among the 21 sequenced *K. pneumoniae* and *E. coli* isolates, all carried β-lactamases; all but one isolate carried at least two β-lactamases (Fig. 3). The most common β-lactamases were *bla*_CTX-M-15_ among *K. pneumoniae* and *bla*_CTX-M-15_ and *bla*_OXA-1_ among *E. coli*. We examined whether β-lactamases differed between mem-S and mem-R isolate pairs from the same cluster. Within the four *K*. *pneumoniae* mem-S and mem-R pairs, there was overlap in β-lactamases, with only a few differences. The isolates in the *E. coli* pair shared the same β-lactamases. Therefore, we looked for other factors that could explain the difference between mem-S and mem-R isolates in the same cluster. Ompk35 was truncated in all *K. pneumoniae* isolates. All mem-S *K. pneumoniae* isolates had intact Ompk36, while all mem-R isolates carried truncated Ompk36. Ompk37 was intact in all *K. pneumoniae* isolates except for one mem-R isolate (Table 3). There was no difference in porins between the mem-S and mem-R isolates in the *E. coli* pair. PBP1, PBP2, and PBP3 (encoded by the genes mrcA, mrdA, and FtsI, respectively) were truncated in all *K. pneumoniae* and *E. coli* isolates, with no difference between mem-S and mem-R isolates (Table 3).

## DISCUSSION

We examined isolates that grew on selective CPE screening plates and found that 60.4% of isolates growing on these plates were non-CPE. We characterized the non-CPE strains. Our three hypotheses were confirmed. All non-CPE isolates were MDR. Non-CPE isolates belonged to multiple FTIR clusters and STs. Non-CPE isolates included both carbapenem-R and carbapenem-S strains. Of 165 non-CP *K. pneumoniae* and *E. coli* isolates, 78.2% were susceptible to meropenem; half of these susceptible isolates had elevated meropenem MICs and all had an MDR phenotype.

We found that the correlation between FTIR and WGS was good for non-CP *K. pneumoniae* but less good for *E. coli*. Meropenem-susceptible and resistant isolates from the same cluster shared some of the same β-lactamases and PBPs but differed in others. Among *K. pneumoniae,* susceptible and resistant isolates in the same cluster differed in the porin ompK36, which was intact in the susceptible isolate and truncated in the resistant strain.

Data about non-CPE growing on selective CPE screening plates come from studies evaluating the performance of various commercial or homemade tests for detecting CPE (8, 16–19). Unlike our study, these previous studies did not aim to characterize the false positives (i.e., non-CPE, either susceptible or resistant to carbapenems) in their samples. In these studies, the percentage of isolates growing on selective CPE screening plates that were non-CPE ranged from 2.5% to 64% (8, 16–19). This highest value, which was comparable to our results, was reported in another study from Israel conducted in 2012–2014. This may reflect similar patient selection for screening, with a low percentage of CPE among screened patients, as well as a high prevalence of ESBL carriage in Israel.

Non-CP CRE are considered to have lower clinical importance than CPE. One reason is that they have less cross-resistance to other antibiotics (9). However, all non-CP CRE in our study were MDR. Most (>70%) were resistant to frequently used antibiotics such as ciprofloxacin, piperacillin/tazobactam, and trimethoprim/sulfamethoxazole.

The ability of non-CPE to grow on selective CPE screening plates apparently stems from a combination of β-lactamases, a porin disruption, and a PBP mutation. All our sequenced non-CPE carried a β-lactamase; all but one isolate carried two to four β-lactamases. The two main porins in *K. pneumoniae* are OmpK35 and OmpK36 (20). In our study, all *K. pneumoniae* isolates had Ompk35 gene disruptions; only those resistant to meropenem also had truncated Ompk36. A recent study examined a global database of porin gene distribution in more than 2,700 *Klebsiella* isolates and found that disruption of both Ompk35 and Ompk36 increased resistance to all β-lactams (21). An additional mechanism that may explain the growth of non-CPE on selective CPE plates is the modification of PBPs, which is known to decrease the binding affinities of β-lactams. Indeed, all the sequenced non-CP isolates in our study had truncated PBPs 1, 2, and 3. Mutant PBPs in *K. pneumoniae* and *E. coli* can result in reduced permeability to some β-lactams including cephalosporins, penicillins, and carbapenems (22).

There are several limitations to this study. First, we did not test gene expression to determine how the disruptions we observed in porins and PBPs affected their functionality. Second, only a small percentage of isolates in our sample underwent WGS.

In conclusion, our study revealed that 60% of isolates growing on CPE selective plates are non-CP Enterobacterales. They represent diverse MDR strains of *K. pneumoniae* and *E. coli* with elevated meropenem MICs. These strains carry multiple mechanisms of resistance, including multiple beta-lactamases and porin mutations. In routine practice, laboratories do not inform physicians of non-CPE/non-CRE detected by screening. However, the knowledge that patients carry such MDR bacteria may be useful to physicians for selecting empiric therapy when signs of infection are present; carriage of MDR organisms is the strongest predictor of MDR infection. Further studies should examine the clinical value of reporting these results.

## Data Availability

Whole genome assemblies are available under BioProject number PRJNA1171162.

## SUPPLEMENTAL MATERIAL

Supplemental material spectrum.02079-24-s0001.docx Tables S1 and S2.

ASM does not own the copyrights to Supplemental Material that may be linked to, or accessed through, an article. The authors have granted ASM a non-exclusive, world-wide license to publish the Supplemental Material files. Please contact the corresponding author directly for reuse.
