# Supplementary material for: Characterization of Enterobacterales growing on selective CPE screening plates with a focus on non-carbapenemase-producing strains
Source: Microbiol Spectr. 2025 Jan 14;13(2):e02079-24. doi: 10.1128/spectrum.02079-24 (PMC11792496; doi:10.1128/spectrum.02079-24)
Supplement: Supplemental material — Tables S1 and S2. [file spectrum.02079-24-s0001.docx]

Supplementary material for " Characterization of Enterobacterales growing on selective CPE screening plates with a focus on non-carbapenemase-producing strains"

**Table S1.** Antibiotic susceptibility (by VITEK) of non-carbapenemase-producing Enterobacterales (N=187).

| **Antibiotic** | **N tested** | **% susceptible** |
| --- | --- | --- |
| Ampicillin | 168 | 0.6 |
| Amoxicillin/ Clavulanic acid | 185 | 6.5 |
| Piperacillin/ Tazobactam | 180 | 3.9 |
| Cefazolin | 185 | 1.6 |
| Cefuroxime | 184 | 0.5 |
| Cefoxitin | 184 | 20.7 |
| Ceftazidime | 184 | 7.1 |
| Ceftriaxone | 177 | 7.9 |
| Meropenem | 187 | 22.5 |
| Ertapenem | 37 | 2.7 |
| Amikacin | 184 | 96.7 |
| Gentamicin | 184 | 67.9 |
| Ciprofloxacin | 184 | 10.9 |
| Fosfomycin | 185 | 71.9 |
| Chloramphenicol | 185 | 35.7 |
| Trimethoprim/ Sulfamethoxazole | 184 | 29.3 |

Table S2. Ertapenem MIC by E-test for non-CP *K. pneumoniae* and *E. coli* isolates.

|  | Non-CP *K. pneumoniae* | Non-CP *E. coli* |
| --- | --- | --- |
| MIC (µg/ml) | N=103  n (cumulative %) | N=62 n (cumulative %) |
| 0.032 | 0 | 0 |
| 0.064 | 0 | 3 (4.8) |
| 0.125 | 2 (1.9) | 4 (11.3) |
| 0.25 | 11 (12.6) | 4 (17.8) |
| 0.5 | 42 (53.4) | 14 (40.4) |
| 1 | 10 (63.1) | 7 (51.7) |
| 2 | 10 (72.8) | 9 (66.2) |
| 4 | 5 (77.7) | 6 (75.9) |
| 8 | 6 (83.5) | 1 (77.5) |
| 12 | 0 | 0 |
| 16 | 4 (87.4) | 0 |
| >=32 | 13 (100) | 11 (95.2) |
| Heteroresistance | 0 | 3 (100) |
